# Supplementary material for: Genome-Wide Identification and Comparative Transcriptome Methods Reveal FaMDHAR50 Regulating Ascorbic Acid Regeneration and Quality Formation of Strawberry Fruits
Source: Int J Mol Sci. 2023 May 30;24(11):9510. doi: 10.3390/ijms24119510 (PMC10253772; doi:10.3390/ijms24119510)
Supplement: Supplementary file 1 [file ijms-24-09510-s001.zip › ijms-2389204-supplementary.pdf]

Supplementary Table S1. Physical and chemical aspects of MDHAR genes in the cultivated strawberry

Rename	Gene Name	Amino Acid	Molecular Weight/KD	*p*I	Instability Index	GRAVY	Subcellular localization
FaMDHADR1	FxaC_4g00110	275	30410.88	9.51	42.37	-0.425	Chloroplast
FaMDHADR2	FxaC_4g06470	275	30419.84	9.46	44.42	-0.422	Chloroplast
FaMDHADR3	FxaC_4g25320	259	28725.2	6.4	32.12	-0.493	mitochondrion
FaMDHADR4	FxaC_4g27160	118	12931.05	5.14	21.89	0.158	Cytoplasm
FaMDHADR5	FxaC_4g33200	222	25096.85	9.15	33.45	-0.264	Chloroplast
FaMDHADR6	FxaC_2g01980	222	25110.88	9.15	34.31	-0.264	Chloroplast
FaMDHADR7	FxaC_2g04540	222	25110.88	9.15	34.31	-0.264	Chloroplast
FaMDHADR8	FxaC_2g17990	118	12944.13	5.38	16.97	0.119	Cytoplasm
FaMDHADR9	FxaC_2g20140	259	28677.22	6.4	32.4	-0.455	mitochondrion
FaMDHADR10	FxaC_2g44500	276	30678.21	9.57	44.74	-0.425	Chloroplast
FaMDHADR11	FxaC_3g13690	118	12960.13	5.37	16.42	0.115	Cytoplasm
FaMDHADR12	FxaC_3g16330	259	28771.29	6.4	31.21	-0.481	mitochondrion
FaMDHADR13	FxaC_3g40000	275	30526.98	9.39	42.23	-0.401	Chloroplast
FaMDHADR14	FxaC_1g07570	222	25123.87	9.03	34.8	-0.264	Chloroplast
FaMDHADR15	FxaC_1g16250	259	28707.26	6.4	30.79	-0.49	mitochondrion
FaMDHADR16	FxaC_1g41620	275	30577.1	9.57	45.03	-0.423	Chloroplast
FaMDHADR17	FxaC_7g01610	221	25182.66	8.52	48.08	-0.34	Chloroplast
FaMDHADR18	FxaC_7g04290	184	20226.55	9.23	48.07	0.057	Chloroplast
FaMDHADR19	FxaC_7g11080	176	19234.88	7.68	51.49	-0.128	Chloroplast
FaMDHADR20	FxaC_7g12730	182	19904.94	8.78	34.37	-0.054	Chloroplast
FaMDHADR21	FxaC_7g27590	498	55740.17	4.98	30.11	-0.359	Chloroplast
FaMDHADR22	FxaC_7g36220	209	23949.18	5.91	42.61	-0.425	Cytoplasm
FaMDHADR23	FxaC_7g38510	377	43029.56	5.52	44.96	-0.214	Cytoplasm
FaMDHADR24	FxaC_7g40110	302	34294.5	7.69	33.99	-0.334	Chloroplast
FaMDHADR25	FxaC_7g40211	433	48732.1	5.15	35.98	-0.373	nucleus
FaMDHADR26	FxaC_7g41490	151	16683.32	5.72	22.99	-0.213	Cytoplasm
FaMDHADR27	FxaC_5g00010	172	19719.36	5.44	42.27	-0.371	Cytoplasm
FaMDHADR28	FxaC_5g08120	498	55913.28	4.95	30.73	-0.366	Chloroplast
FaMDHADR29	FxaC_5g23910	182	19890.92	8.78	34.37	-0.055	Chloroplast
FaMDHADR30	FxaC_5g26330	177	19335.93	7.68	52.04	-0.151	Chloroplast
FaMDHADR31	FxaC_5g36880	182	19987.23	9.1	50.53	0.068	Chloroplast
FaMDHADR32	FxaC_5g39640	226	25331.85	8.5	48.51	-0.304	Chloroplast
FaMDHADR33	FxaC_5g41320	226	25331.85	8.5	48.51	-0.304	Chloroplast
FaMDHADR34	FxaC_8g03420	498	55831.22	4.95	31.21	-0.377	Chloroplast
FaMDHADR35	FxaC_8g06690	186	21329.73	6.92	24.49	-0.385	Cytoplasm
FaMDHADR36	FxaC_8g08230	428	47936.11	5.26	32.17	-0.39	Chloroplast
FaMDHADR37	FxaC_8g08350	302	34245.38	7.68	35.72	-0.35	Chloroplast
FaMDHADR38	FxaC_8g09691	377	43001.46	5.52	44.18	-0.222	Cytoplasm
FaMDHADR39	FxaC_8g11780	209	23905.08	5.74	43.75	-0.411	Cytoplasm
FaMDHADR40	FxaC_8g23960	182	20013.09	8.78	31.87	-0.059	Chloroplast

FaMDHADR41	FxaC_8g33410	209	23209.89	8.52	44.53	0.025	Chloroplast
FaMDHADR42	FxaC_6g01990	226	25522.16	8.24	47.18	-0.259	Chloroplast
FaMDHADR43	FxaC_6g06010	181	19902.13	9.1	48.62	0.046	Chloroplast
FaMDHADR44	FxaC_6g15390	177	19307.89	6.57	48.26	-0.134	Chloroplast
FaMDHADR45	FxaC_6g17860	182	19938.92	8.62	33.64	-0.058	Chloroplast
FaMDHADR46	FxaC_6g32150	498	55865.29	5.01	29.62	-0.367	Vacuole
FaMDHADR47	FxaC_6g35610	131	14678.39	5.65	31.26	-0.434	Chloroplast
FaMDHADR48	FxaC_6g40070	209	23972.18	6.18	43.18	-0.458	Cytoplasm
FaMDHADR49	FxaC_6g41931	380	43420.03	5.71	44.87	-0.204	Cytoplasm
FaMDHADR50	FxaC_6g42700	122	13342.55	5.42	20.12	0.041	Cytoplasm
FaMDHADR51	FxaC_6g43960	302	34212.26	6.92	35.21	-0.344	Chloroplast
FaMDHADR52	FxaC_12g12460	146	16402.85	5.11	22.49	-0.116	Vacuole
FaMDHADR53	FxaC_12g13220	568	63193.99	4.62	33.65	-0.348	Endoplasmic reticulum
FaMDHADR54	FxaC_12g26740	479	53493.78	6.93	44.03	-0.249	Plasma membrane
FaMDHADR55	FxaC_12g29200	185	19761.96	9.44	49.93	0.12	Chloroplast
FaMDHADR56	FxaC_12g31420	691	75046.72	8.88	51.64	-0.406	nucleus
FaMDHADR57	FxaC_12g37520	200	21815.81	8.75	43.88	-0.242	Chloroplast
FaMDHADR58	FxaC_12g48920	292	32673.09	8.42	53.07	-0.477	Cytoplasm
FaMDHADR59	FxaC_10g05050	455	50508.03	6.14	56.17	-0.39	Chloroplast
FaMDHADR60	FxaC_10g10460	489	53000.68	5.32	34.57	-0.199	nucleus
FaMDHADR61	FxaC_10g13810	185	20483.19	6.41	45.94	-0.341	nucleus
FaMDHADR62	FxaC_10g19610	215	23031.78	9.54	47.24	0.101	Chloroplast
FaMDHADR63	FxaC_10g24260	479	53443.77	6.75	42.72	-0.238	Plasma membrane
FaMDHADR64	FxaC_10g36730	568	63203.94	4.61	34.98	-0.354	Chloroplast
FaMDHADR65	FxaC_10g37390	146	16403.94	5.41	25.43	-0.125	Chloroplast
FaMDHADR66	FxaC_11g11180	209	22839.88	8.42	51.17	-0.308	nucleus
FaMDHADR67	FxaC_11g11390	200	21815.81	8.75	43.88	-0.242	Chloroplast
FaMDHADR68	FxaC_11g17320	687	74524.08	8.88	53.47	-0.394	nucleus
FaMDHADR69	FxaC_11g19320	185	19797.99	9.44	47.85	0.14	Chloroplast
FaMDHADR70	FxaC_11g21770	479	53650.2	7.75	42.59	-0.234	Cytoplasm
FaMDHADR71	FxaC_11g33510	570	63592.62	4.67	32.45	-0.344	Chloroplast
FaMDHADR72	FxaC_11g34620	146	16403.88	5.25	21.91	-0.165	Chloroplast
FaMDHADR73	FxaC_9g15860	163	undefined	undefined	23.99	0.091	undefined
FaMDHADR74	FxaC_9g17060	566	63185.07	4.68	33.83	-0.338	Chloroplast
FaMDHADR75	FxaC_9g30810	479	53598.98	6.93	42.72	-0.249	Cytoplasm
FaMDHADR76	FxaC_9g36200	692	74944.5	8.87	52.74	-0.404	nucleus
FaMDHADR77	FxaC_9g44760	489	52949.46	5.26	37.11	-0.216	nucleus
FaMDHADR78	FxaC_16g06530	174	19546.32	8.91	51.44	-0.273	Chloroplast
FaMDHADR79	FxaC_16g26590	504	56335.15	8.05	44.45	-0.236	Chloroplast
FaMDHADR80	FxaC_16g28890	569	64011.8	5.43	36.07	-0.287	Cytoplasm
FaMDHADR81	FxaC_16g30330	131	14884.97	6.96	29.69	-0.573	Cytoplasm

FaMDHADR82	FxaC_15g00630	1081	121777.26	5.5	46.78	-0.351	Vacuole
FaMDHADR83	FxaC_15g01670	131	14919.97	6.9	34.59	-0.583	Cytoplasm
FaMDHADR84	FxaC_15g03231	569	64041.81	5.34	38.22	-0.273	Cytoplasm
FaMDHADR85	FxaC_15g37130	400	44709.72	5	34.22	-0.392	nucleus
FaMDHADR86	FxaC_13g02080	131	14897.02	6.96	30.34	-0.534	Cytoplasm
FaMDHADR87	FxaC_13g04030	567	63848.57	5.3	36.48	-0.279	Cytoplasm
FaMDHADR88	FxaC_13g04040	478	54042.56	4.96	43.4	-0.343	Cytoplasm
FaMDHADR89	FxaC_13g06350	504	56335.15	8.05	44.45	-0.236	Chloroplast
FaMDHADR90	FxaC_13g47690	185	20672.61	8.4	45.75	-0.196	Chloroplast
FaMDHADR91	FxaC_14g00890	1078	121620.91	5.58	46.56	-0.362	Chloroplast
FaMDHADR92	FxaC_14g01980	131	14919.93	6.42	33.69	-0.639	Cytoplasm
FaMDHADR93	FxaC_14g03470	478	54118.6	4.96	45.57	-0.356	cytoskeleton
FaMDHADR94	FxaC_14g03471	569	63668.32	5.37	36.18	-0.296	Cytoplasm
FaMDHADR95	FxaC_14g05600	504	56275.06	8.05	42.74	-0.239	Chloroplast
FaMDHADR96	FxaC_14g36530	185	20677.68	8.96	40.56	-0.165	Chloroplast
FaMDHADR97	FxaC_17g19470	246	27154.29	8.63	43.85	-0.217	Cytoplasm
FaMDHADR98	FxaC_17g22780	111	12165.17	9.61	25.96	0.068	Chloroplast
FaMDHADR99	FxaC_17g45150	490	54714.28	5.84	32.44	-0.265	Plasma membrane
FaMDHADR100	FxaC_20g15330	267	29309.8	7.16	43.32	-0.149	Cytoplasm
FaMDHADR101	FxaC_20g18530	419	46480.08	6.45	45.61	-0.429	Chloroplast
FaMDHADR102	FxaC_20g33140	125	14101.42	9.15	19.15	-0.137	Cytoplasm
FaMDHADR103	FxaC_20g35030	450	50244.16	5.3	33.6	-0.186	Vacuole
FaMDHADR104	FxaC_20g35040	93	9903.43	5.42	23.22	0.523	Extracellular
FaMDHADR105	FxaC_18g12360	450	50112.99	5.13	33.93	-0.167	Vacuole
FaMDHADR106	FxaC_18g27580	364	40386.97	5.27	33.71	-0.378	cytoskeleton
FaMDHADR107	FxaC_18g31080	267	29388.98	6.89	42.52	-0.1	Cytoplasm
FaMDHADR108	FxaC_18g31950	123	13789.59	8.93	33.29	-0.52	mitochondrion
FaMDHADR109	FxaC_19g12830	253	28477.96	6.79	41.67	-0.203	Cytoplasm
FaMDHADR110	FxaC_19g15790	364	40250.71	5.33	37.22	-0.41	cytoskeleton
FaMDHADR111	FxaC_19g32240	450	50114.96	5.13	33.22	-0.176	Vacuole
FaMDHADR112	FxaC_19g32320	450	50000.86	5.17	31.68	-0.161	Vacuole
FaMDHADR113	FxaC_21g00310	147	16523.82	5.45	33.13	-0.357	Chloroplast
FaMDHADR114	FxaC_21g15180	437	47922.89	5.65	34.11	-0.185	Extracellular
FaMDHADR115	FxaC_21g29330	531	59745.45	5.12	36.21	-0.112	Chloroplast
FaMDHADR116	FxaC_21g37320	527	58037.82	6.4	46.9	-0.296	Chloroplast
FaMDHADR117	FxaC_21g41350	190	22064.59	8.7	53.24	-0.275	Cytoplasm
FaMDHADR118	FxaC_21g48600	131	14628.33	5.65	34.58	-0.456	mitochondrion
FaMDHADR119	FxaC_21g59240	139	15399.6	5	19.99	-0.174	Chloroplast
FaMDHADR120	FxaC_21g62360	379	41553.4	6.42	27.16	-0.319	Cytoplasm
FaMDHADR121	FxaC_21g70020	187	20325.27	8.79	32.51	-0.097	Chloroplast
FaMDHADR122	FxaC_23g05100	93	10329.15	9.03	16.74	-0.127	Cytoplasm
FaMDHADR123	FxaC_23g07430	531	59855.43	5.08	37.11	-0.148	Chloroplast
FaMDHADR124	FxaC_23g13770	178	19386.45	8.48	43.82	0.058	Chloroplast

FaMDHADR125	FxaC_23g13790	110	12123.02	5.56	23.99	0.005	Chloroplast
FaMDHADR126	FxaC_23g16400	521	57245.98	6.14	43.52	-0.255	Chloroplast
FaMDHADR127	FxaC_23g18700	113	13167.33	6.28	45.84	-0.304	Cytoplasm
FaMDHADR128	FxaC_23g25200	131	14610.31	5.41	32.31	-0.415	Chloroplast
FaMDHADR129	FxaC_23g34040	139	15427.65	5	19.45	-0.157	Chloroplast
FaMDHADR130	FxaC_23g36330	382	41836.88	7.47	25.86	-0.28	Cytoplasm
FaMDHADR131	FxaC_23g43450	186	20266.25	8.79	30.56	-0.08	Chloroplast
FaMDHADR132	FxaC_23g49980	132	14683.69	5.73	25.61	-0.33	Cytoplasm
FaMDHADR133	FxaC_23g57730	440	48209.26	5.46	34.79	-0.163	Extracellular
FaMDHADR134	FxaC_22g05430	132	14645.66	5.62	32.31	-0.164	Cytoplasm
FaMDHADR135	FxaC_22g05431	134	14883.98	5.37	31.56	-0.12	Cytoplasm
FaMDHADR136	FxaC_22g13040	117	13105.3	9.41	31.09	0.194	Extracellular
FaMDHADR137	FxaC_22g24970	531	59801.37	5.05	35.67	-0.144	Chloroplast
FaMDHADR138	FxaC_22g31480	178	19329.36	8.84	46.64	0.008	Chloroplast
FaMDHADR139	FxaC_22g34070	521	57366.13	6.3	46.11	-0.276	Chloroplast
FaMDHADR140	FxaC_22g36670	185	21492.81	8.84	52.89	-0.423	Cytoplasm
FaMDHADR141	FxaC_22g50040	131	14642.36	5.42	29.52	-0.432	Chloroplast
FaMDHADR142	FxaC_22g58990	139	15427.65	5	19.45	-0.157	Chloroplast
FaMDHADR143	FxaC_22g61300	382	41974.1	7.47	26.38	-0.292	Cytoplasm
FaMDHADR144	FxaC_22g72010	186	20313.32	9.1	36.17	-0.146	Chloroplast
FaMDHADR145	FxaC_24g05470	191	20869.92	9.18	33.24	-0.153	Chloroplast
FaMDHADR146	FxaC_24g11100	382	42010.05	6.86	25.66	-0.303	Cytoplasm
FaMDHADR147	FxaC_24g13440	139	15371.54	4.86	25.65	-0.152	Chloroplast
FaMDHADR148	FxaC_24g21760	131	14740.51	6.18	28.05	-0.502	Chloroplast
FaMDHADR149	FxaC_24g28300	185	21475.81	8.84	53.56	-0.418	Cytoplasm
FaMDHADR150	FxaC_24g30510	523	57537.29	6.37	45.22	-0.285	Chloroplast
FaMDHADR151	FxaC_24g33600	178	19398.47	9.1	49.85	-0.001	Chloroplast
FaMDHADR152	FxaC_24g33970	178	19398.47	9.1	49.85	-0.001	Chloroplast
FaMDHADR153	FxaC_24g40460	531	59728.35	5.12	36.8	-0.13	Chloroplast
FaMDHADR154	FxaC_24g50750	437	47916.9	5.57	34.81	-0.181	Chloroplast
FaMDHADR155	FxaC_24g58200	136	14900.93	5.37	33.01	-0.171	Cytoplasm
FaMDHADR156	FxaC_27g04320	188	21357.5	6.12	40.3	-0.252	Chloroplast
FaMDHADR157	FxaC_27g31110	199	22143.41	9.28	49.47	-0.202	Chloroplast
FaMDHADR158	FxaC_27g34950	195	21797.12	9.28	50.53	-0.159	Chloroplast
FaMDHADR159	FxaC_27g42010	142	15616.68	4.91	34.2	-0.149	Cytoplasm
FaMDHADR160	FxaC_25g04940	188	21302.51	6.44	39.16	-0.232	Chloroplast
FaMDHADR161	FxaC_25g36940	196	21873.12	8.96	55.54	-0.163	Chloroplast
FaMDHADR162	FxaC_25g46170	142	15490.65	5.03	33.7	-0.058	Cytoplasm
FaMDHADR163	FxaC_26g18530	195	21960.34	9.15	46.1	-0.125	Chloroplast
FaMDHADR164	FxaC_26g23660	284	31099.94	8.44	33.88	-0.059	Extracellular
FaMDHADR165	FxaC_26g37720	188	21280.4	5.95	36.51	-0.224	Chloroplast
FaMDHADR166	FxaC_28g07990	142	15658.81	5.07	29.72	-0.186	Chloroplast
FaMDHADR167	FxaC_28g17230	195	21864.16	9.11	49.93	-0.161	Chloroplast
FaMDHADR168	FxaC_28g35170	188	21290.62	6.29	47.71	-0.196	Chloroplast

Supplementary Table S2. Gene quantitative primers

Genes	Sequences (5′-3′)
*35sFa*MDHAR*50*	*35s::Fa*MDHAR50-F:
	ACGAGCTCGGATCCTCTAGAATGCTTCAGGCAATGGAGAG
	*35s::Fa*MDHAR50-*R*:
	TCGTCCTTGTAGTCAGATCTGCTGCTCTTTGGATGGGTTTG
Actin2	Actin-F:GGGTTTGCTGGAGATGAT
	Actin-R:CAGTTAGGAGAACTGGGTGC
*Fa*AO	*Fa*AO-F: CCTACATCACTGCTTCTCCAC
	*Fa*AO-R: TGTGAGAAGCAATGGCTCG
*Fa*APX	*Fa*APX-F: GGCCAAGAGGAAGCTCAGAG
	*Fa*APX-R: CTTCATAGTTCCGAATGGGCC
*Fa*ANS	*Fa*ANS-F: GGAGTACGTGAGACCCGAAG
	*Fa*ANS-R: CTACATTTCTCCCTCACCTTGATG
FaCEL1	*FaCEL1-F*: GGATGAAGAATGCGAATCAATGG
	FaCEL1-R: TCAGTACTGTATCCAGGCCAC
*Fa*DHAR	*Fa*DHAR-F: GACCTGTCACTTGCTCCCAA
	*Fa*DHAR-R:AGGGAATCTGGAACCGACC
*Fa*F3′5′H	*Fa*F3′5′H-F:GGAGAACAACAATAATAATCATGAAGATGGT
	*Fa*F3′5′H-R:TCTCTGTTAGTTCTTCTTGAACTCTTCTC
*Fa*GaLUR	*Fa*GaLUR-F: GCAATTAGAGCTGGTTACCGACA
	*Fa*GaLUR-R: GAGATCTTTCTCCGAGCCG
*Fa*GalLDH	*Fa*GLDH-F:GCTGGGATCAGGGTCCAG
	*Fa*GLDH-R:CCCTAATGGATGCAAAGTTCTGC
*Fa*MDHAR	FaMDHAR-F:GACCTTGGGAAACTGGAGATG
	*Fa*MDHAR-R: TCAGTCTACAGCTCTTCCTGC
*Fa*NCED1	*Fa*NCED1-F:GAGGAAGCTTGTTCTCCGAG
	*Fa*NCED1-R:AACGTCTTCATTTCTTGGTAGGTG
FaSPS	*FaSPS*-F: AAGATGCAGGCACAATGGAC
	FaSPS-R: TTGTGGCTGCCTTGGTTTG
FaSNS	*FaSNS*-F: AATGAACCGTGCGCGTAATG
	FaSNS-R: ACAACAGTGAGGCCAAAAGC
